# The Effects of Heart Rate Monitoring on Ratings of Perceived Exertion and Attention Allocation in Individuals of Varying Fitness Levels

**DOI:** 10.3389/fspor.2021.798941

**Published:** 2022-01-07

**Authors:** Robyn Braun-Trocchio, Ashlynn Williams, Kaitlyn Harrison, Elizabeth Warfield, Jessica Renteria

**Affiliations:** Sport and Exercise Psychology Lab, Kinesiology Department, Texas Christian University, Fort Worth, TX, United States

**Keywords:** RPE, perceived effort, HR, wearable fitness device, physical activity, exercise psychology

## Abstract

There has been a rapid increase in the use of wearable technology-based physical activity trackers. Most of these physical activity trackers include tracking and displaying the individual's heart rate (HR). There is little known about how HR monitoring influences the perception of exertion and attention allocation. Shifting attentional focus toward the body (association), such as monitoring HR, instead of environmental stimuli (dissociation) may increase one's perceived level of exertion. The purpose of the study was to examine the effects of HR monitoring on ratings of perceived exertion (RPE) and attention allocation during an exertive stepping task in individuals of varying fitness levels. The YMCA stepping task normative values determined fitness levels. For the experimental condition, participants were randomly assigned to one of two conditions (i.e., HR monitoring or control) and completed a stepping task with a weighted vest at 20% of their bodyweight. HR, RPE, and attention allocation were collected at 30-s intervals. Performing the stepping task resulted in a gradual increase of HR and RPE along with a shift from dissociative to associative attention across all conditions. Monitoring one's HR during the task resulted in more dissociative attention allocation, however, no RPE differences were reported between the two conditions. Unfit individuals reported lower levels of RPE during the first time point compared to fit individuals despite having higher HR throughout the task. The results of this study have relevance for applied practitioners implementing physical activity interventions with individuals who monitor their HR.

## Introduction

There has been a rapid increase in the use of wearable fitness devices (WFD) with new products released to the consumer market every year. Wearable technology is an electronic device that can be worn on the body (e.g., watch) or on clothing (Wright and Keith, 2014). Most of these physical activity (PA) devices include tracking and displaying the individual's heart rate (HR) along with other PA data such as acceleration of individual's movement and calories burned (Bloss, 2017; Nazari et al., 2017). In the United States, the wearable industry was valued at $18 million in 2018 and is expected to reach $64 million by 2023 (Loomba and Khairnar, 2018). More specifically, worldwide wearable device vendors distributed a total of 125.5 million devices, which is an increase from the 104.3 million units distributed in 2016, making a 20.4% growth (Jia et al., 2018). The majority of research on wearable technology has focused on the accuracy and reliability of these devices (Nazari et al., 2017; Shah et al., 2017; Hernando et al., 2018) as well as promoting PA (Chiauzzi et al., 2015; Jo et al., 2019).

With the continued rise of the obesity epidemic and physical inactivity, WFD are being utilized as behavioral interventions to promote PA. The World Health Organization (2020) recommends 150-min of moderate intensity PA or 75-min of vigorous PA a week to reduce the risk of developing chronic diseases such as cardiovascular disease and type II diabetes. One way to measure the intensity of PA is by measuring an individual's HR which can be done through a WFD. For moderate intensity PA, the target HR should be between 64 and 76% maximal HR while vigorous intensity is between 77 and 93% maximal HR (Riebe et al., 2018). HR responses to exercise is moderated by knowledge about the suggested intensity and feedback in meeting those levels (Blanchard et al., 1974). HR monitoring has shown to increase overall daily activity and the amount of time in moderate-to-vigorous PA compared to not receiving the HR feedback (McManus et al., 2008).

Another way to measure PA intensity is through Borg's ratings of perceived exertion (RPE) scale. Perceived exertion during exercise, is a psychophysiological term defined as the subjective intensity of effort, strain, discomfort, and/or fatigue (Noble and Robertson, 1996). Borg (1982) developed a 15-point scale ranging from 6 to 20. These range in ratings from 6 perceiving “no exertion at all” to 20 perceiving a “maximal exertion” of effort. The RPE scale corresponds to the HR range of a normal healthy young male (60–200 beats/min) and HR is predicted by multiplying an individual's RPE score by 10. However, actual HR can vary depending on age and physical condition. This scale represents a valid and reliable measurement of perceptual effort intensity (Noble and Robertson, 1996). RPE is often used for determining appropriate levels of exercise intensity to define the cardiorespiratory training zone for an individually prescribed exercise regimen. Monitoring RPE levels assists individuals in adjusting their intensity levels. Since there is only a correlational relationship between RPE and HR, more research is needed to determine if HR directly influences perception of effort during increased PA intensity (Hampson et al., 2001). Furthermore, the research between fitness levels and RPE is equivocal. Some research reports that fit individuals report lower RPE values (Hassmen, 1990; Travlos and Marisi, 1996) or no differences (Parfitt et al., 1996; Faulkner and Eston, 2007; Faulkner et al., 2007). More specifically, there is little known about how HR monitoring through a WFD influences an individual's perception of exertion and attention allocation.

There are two categories of attention allocation, associative strategies which help direct an individual's focus internally, and toward somatic cues such as attending to breathing, while dissociative strategies direct one's focus externally such as daydreaming, random, or intentional thoughts (Masters and Ogles, 1998; Scott et al., 1999; Tenenbaum, 2005). According to Tenenbaum's (2001) effort-related model, individuals tend to shift attention as a function of increased physical workload across all PA settings. As physical workload intensity increases, individuals tend to alternate their attentional focus from a dissociative style to an associative one occurring around an RPE of 13 (“somewhat hard”) (Welch et al., 2007). Research has demonstrated that associative strategies allow for smaller adjustments of effort by monitoring the physiological cues while dissociative strategies divert attention away from the physiological cues thus reducing the perceptions of exertion (Lind et al., 2009; Rose and Parfitt, 2010). Consequently, shifting attentional focus toward the body (association), such as monitoring HR, instead of environmental stimuli (dissociation) may increase one's perceived level of exertion.

The purpose of the current study was to investigate the effects of HR monitoring on the perception of exertion and attention allocation during an exertive stepping task with individuals of varying fitness levels. The first hypothesis was that individuals in the HR monitoring condition would have higher RPE levels and associative attention allocation compared to the control condition without HR monitoring. The second hypothesis was that unfit individuals would have higher RPE and associative attention allocation compared to fit individuals. Finally, it was hypothesized that unfit individuals with HR monitoring would have the highest RPE and most associative attention allocation.

## Materials and Methods

### Participants

A total of 66 participants completed the study (females = 47 and males = 19) ranging in age from 18 to 50 (*M* = 21.97, *SD* = 6.18). Participants were randomly assigned to either a HR monitoring condition by wearing a wrist watch (*n* = 35) or a control/no monitoring (*n* = 31) condition. The General Health and Life Type Questionnaire—Shortened Version (GHLQ; British Columbia Department of Health, 1975) assessed aspects of personal health history. Only participants who did not have any physical or psychological disabilities that would interfere with the completion of an exertive stepping task were included in the study. Using the standardized 3-min YMCA stepping protocol participants were categorized as fit (*n* = 40) and unfit (*n* = 26).

### Physical Task

The stepping task was adapted from the YMCA stepping protocol (Braun and Tenenbaum, 2018). The adapted step task was selected since it is a novel, yet familiar, task to most participants limiting the amount of biases toward the activity, which can influence an individual's RPE. A similar stepping task has been used to examine VO_2_ max (Santo and Golding, 2003) and exercise adherence (Glaros and Janelle, 2001). For the actual stepping task, the participants stepped up and down in cadence with a metronome at 96 beats per minute on to the Rogue Resin Plyo Box (height = 30.5 cm). If participants deviated from the pace, researchers promptly corrected them to return to the set cadence. Baseline testing for each participant included completing the standardized YMCA 3-min stepping protocol to determine fitness level. Following a 1-min rest, HR was taken and the recovery HR was compared to the normative values based on the participant's age and gender (Golding et al., 1989). According to the normative values, fitness levels ranged from very poor to excellent. Fit individuals were categorized as being average or above and unfit individuals were categorized as below average or below. Following the fitness test, the adapted experimental stepping task was completed. First, to manipulate exercise intensity a weighted vest corresponding to 20% of the individual's body weight was added to the participant. Then, each participant completed the experimental task until volitional fatigue or the participant no longer maintained the cadence.

### Instrumentation

#### Demographic Questionnaire

This questionnaire includes demographic information such as participant's age, gender, and frequency and intensity of regular physical activity.

#### Health History Form

The GHLQ assessed aspects of personal health history including more specific items on coronary and cardiovascular conditions (i.e., heart murmur, irregular heartbeat, and high blood pressure), respiratory (i.e., asthma), and other diseases or ailments (i.e., orthopedic injuries) (British Columbia Department of Health, 1975).

#### Task-Specific Motivation Scale

This questionnaire is designed to examine task-specific self-efficacy (two items based on Bandura's, 1977, self-efficacy measurement guidelines), task-specific perceived ability, and task-specific motivation (Hutchinson and Tenenbaum, 2007). Participants rated their task-specific self-efficacy, perceived ability, and motivation on a Likert-type scale ranging from 0 (*very low*) to 10 (*very high*).

#### Commitment Check

This scale asks participants to report their commitment and effort investment on a 10-point Likert-type scale ranging from 1 (*none/not at all*) to 10 (*very much/very well*) at the end of the task. The scale includes three items: (a) “How hard did you try while you were completing this task?” (b) “How well do you believe you handled any physical discomfort or pain during the task?” and, (c) “How much effort did you invest in the task?”.

#### Rating of Perceived Exertion

The RPE scale is a 15-point category-ratio scale ranging from 6 (*no exertion at all)* to 20 (*maximal exertion*) measuring perceived exertion during the exercise task (Borg, 1982). The higher the RPE score, the higher the rating of perceived exertion. This scale has high test-retest reliability (*r* > 0.83) and is closely related to various physiological and chemical measurements (Borg, 1982, 1998).

#### Attention

To measure attention allocation during the task, a 10-point scale ranging from 0 (*external thoughts, daydreaming, environment, singing songs*) to 10 (*internal thoughts, how body feels, breathing, muscles)* was used (Tammen, 1996). During physical exertion, the one-question scale is an effective and valid measure of attention strategy (Tammen, 1996).

### Apparatus

#### Metronome

To keep the cadence during the stepping task and maintain the required number of steps, a Steinway and Sons Metronome App from Apple was utilized. The metronome app was set to 96 beats per minute, which elicited 24 completed cycles of stepping or 96-foot strikes per minute.

#### Heart Rate Monitor

HR was measured using Polar's H10 HR monitor system via chest worn sensor strap and a wrist watch HR receiver unit.

#### Weighted Vest

The Rogue Fitness MiR Pro Weighted Vest was utilized to increase the load to 20% of the individuals body weight. The vest was adjusted to fit the participant.

### Procedure

Preceding any data collection, IRB approval was granted. Participants refrained from physical activity for at least 24 h prior to testing. To reduce possible social facilitation effect, participants were tested individually in a private room. Participants were given an informed consent form outlining the purpose, format, and tasks of the study. Upon agreement to participate in the study, participants completed the demographic, and GHLQ to determine if they were a viable candidate. Only participants who did not have any physical or psychological disabilities that would interfere with the completion of an exertive stepping task were included in the study. All data collection occurred in a single session.

First, participants were randomly assigned to one of the two conditions (i.e., HR monitoring or control). During the experimental task, participants in the HR monitoring condition wore a watch which displayed their HR. In the control condition, the researcher recorded the participant's HR without their knowledge.

Then, participant's height and weight were measured. Next, the researchers attached the Polar HR device to the participant and baseline HR measurements were collected for 3-min. After baseline HR, the RPE and attention scales were explained using a standard script. If the participant was unsure about any of the scales, the researcher offered clarification. Resting attention, RPE and HR were recorded.

Next, the participants were familiarized with the stepper and the metronome and provided with the opportunity to practice the stepping task. Familiarization included the participant taking eight unweighted steps up and down onto the Rogue Resin Plyo Box paced by a metronome at 96 beats per minute while connected to a HR monitor. After participants felt comfortable with the task, the task-specific motivation scale was administered.

Following familiarization, participants completed the 3-min YMCA stepping task to determine the individual's fitness levels. At 30-s intervals throughout the task, participants were asked to verbally state their RPE and attention number and the researcher recorded their HR without letting the participant know. The HR was taken 1-min post-exercise and the recovery HR was compared to the normative values based on the participant's age and gender (Golding et al., 1989). Participants were grouped as fit or unfit based on the normative values.

After participant's HR had returned to resting levels (*M* = 337.52-s, *SD* = 22.71-s), the participant was fitted with the weighted vest corresponding to a load of 20% of the participant's body weight. Baseline RPE, attention, and HR was taken prior to beginning the task. Participants then completed the adapted YMCA stepping task (Braun and Tenenbaum, 2018) with the weighted vest until volitional fatigue or the participant no longer maintained the cadence. RPE and attention, were collected from the participants at 30-s intervals throughout the task. HR was also collected at 30-s intervals. Participants in the HR monitoring condition looked at the watch and verbally stated their HR to the researcher when asked. Participants in this condition were told to only check the HR monitor when requested by the researcher. For the participants in the control condition, the researcher recorded their HR without their knowledge. Upon completion of the task, participants completed a commitment check and were debriefed.

### Data Analysis

Multivariate Analysis of Variance (MANOVA) was utilized for the motivation and commitment check items. To test the hypotheses, a repeated measure Analysis of Variance (RM ANOVA) was used for RPE and attention allocation through increments of physical effort expenditure. The two conditions (i.e., HR monitoring and control) and two fitness levels (i.e., fit and unfit) were considered between subject factors and time interval (categorized in 30-s intervals) was considered the within subject repeated factor. When Mauchly's sphericity reached significance for the main effects (*p* < 0.05) then the Greenhouse-Geisser (GG) epsilon correction coefficient was implemented. Partial eta squared (*η_p_*) was used as a measure of effect size.

## Results

### Attrition

Descriptive examination indicated high participant attrition rate following the 4th time interval (i.e., 2-min). After this time point, ~11% of participants ceased the stepping task reducing the power of detecting an effect. Thus, the 4th interval was set as the cut-off point in the present analysis.

### Task Specific Motivations

To test pre-task differences in motivation between the conditions (HR monitoring) and fitness level (fit and unfit), a MANOVA was conducted. Results revealed a non-significant condition effect, Wilk's λ = 0.977, *F*_(4,59)_ = 0.354, *p* > 0.05, *η_p_* = 0.023, non-significant fitness effect, Wilk's λ = 0.911, *F*_(4,59)_ = 1.43, *p* > 0.05, *η_p_* = 0.089, and a non-significant interaction effect, Wilk's λ = 0.967, *F*_(4,59)_ = 0.504, *p* > 0.05, *η_p_* = 0.033. On average, participants were highly self-efficacious about their ability to perform well (*M* = 8.54, *SD* = 0.23).

### Commitment Check

Results from a MANOVA reported no significant differences among the conditions, *Wilk's* λ = 0.877, *F*_(3,60)_ = 2.80, *p* > 0.05, *η_p_* = 0.123, fitness levels, *Wilk's* λ = 0.928, *F*_(3,60)_ = 1.56, *p* > 0.05, *η_p_* = 0.072, and interaction, *Wilk's* λ = 0.987, *F*_(3,60)_ = 0.26, *p* > 0.05, *η_p_* = 0.013. In general, participants reported high commitment levels to the stepping task (*M* = 4.56, *SD* = 0.26).

### Ratings of Perceived Exertion

**Figure 1:**
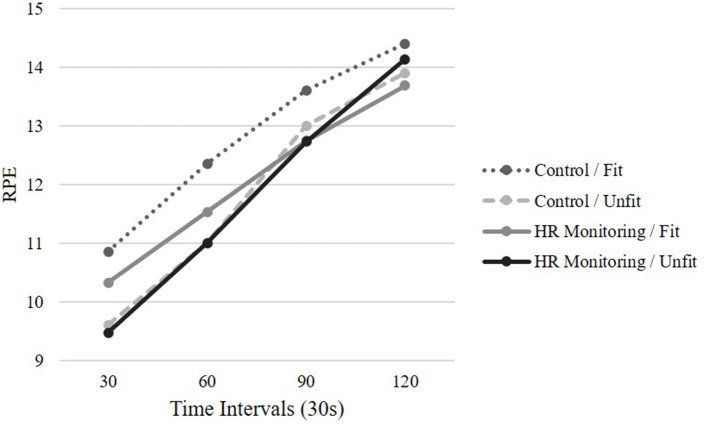
Mean RPE across time by condition and fitness level.

A 4 (time intervals) by 2 (conditions) by 2 (fitness) RM ANOVA was conducted for RPE. A significant main effect for time was revealed GG_ms_ = 289.00, *F*_(1.88,112.55)_ = 193.20, *p* < 0.001, *η_p_* = 0.76, indicating that as time progressed participants RPE increased in the conditions. A significant time by fitness interaction was found GG_ms_ = 5.81, *F*_(1.88,112.55)_ = 3.88, *p* = 0.026, *η_p_* = 0.061. Follow-up t-tests revealed the difference between fitness levels was significant at the 30-s time point, *t*_(64)_ = 2.115, *p* = 0.038 with fit individuals (*M* = 3.90, *SD* = 2.84) reporting higher levels of RPE compared to the unfit group (*M* = 2.80, *SD* = 2.41). Analysis revealed no significant effects for time by condition, GG_ms_ = 0.446, *F*_(1.88,112.55)_ = 0.298, *p* > 0.05, *η_p_* = 0.005, or time by condition by fitness, GG_ms_ = 351, *F*_(1.88,112.55)_ = 0.235, *p* > 0.05, *η_p_* = 0.004 (see Figure 1).

### Attention Allocation

**Figure 2:**
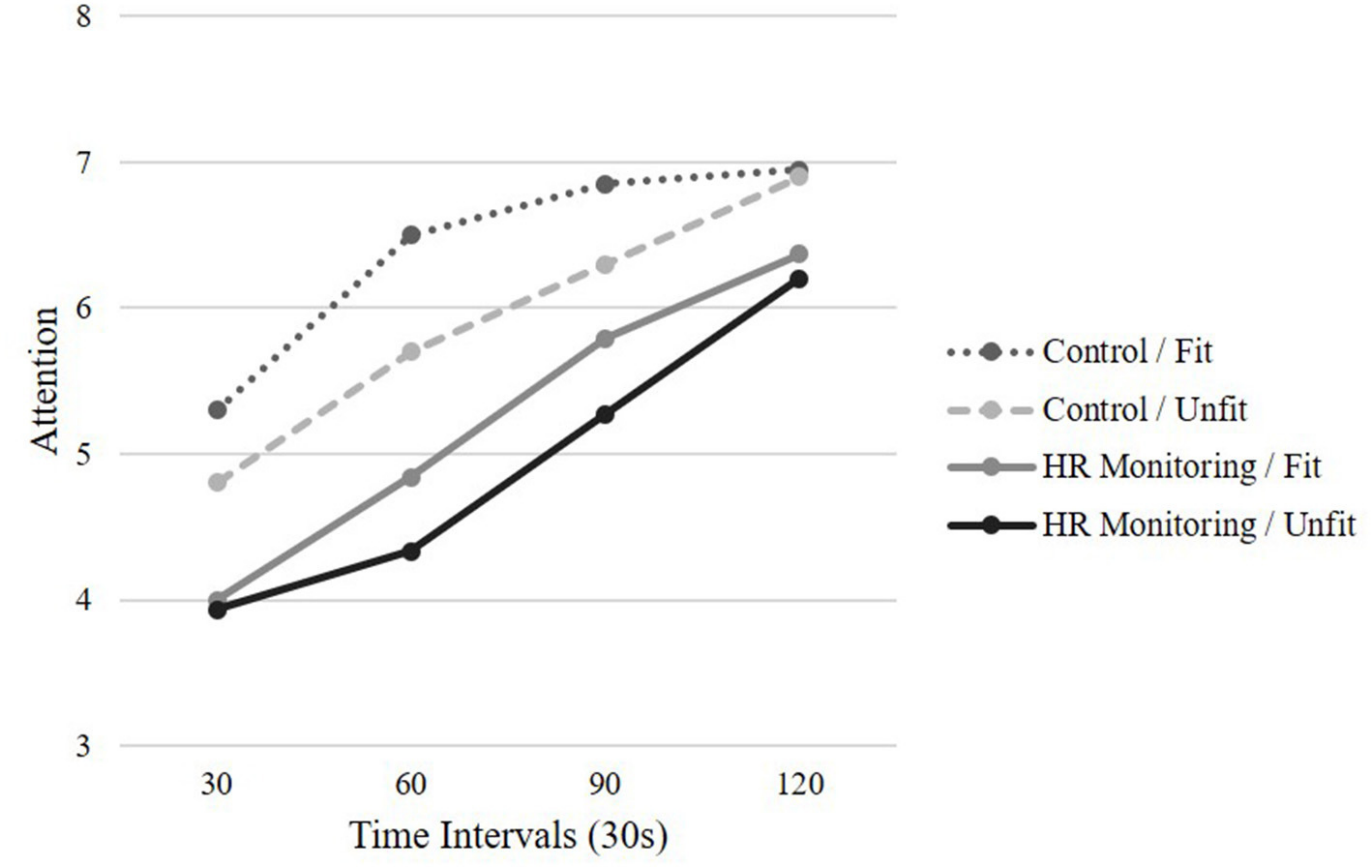
Mean attention across time by condition and fitness level.

The analysis conducted on attention was similar to the one conducted on RPE. A significant main effect of time was revealed, GG_ms_ = 77.470, *F*_(1.89,113.55)_ = 51.55, *p* < 0.001, *η_p_* = 0.462. This suggests that as the task duration increased attention shifted from dissociative to associative. The main effect for condition was not significant, *F*_(1,60)_ = 3.42, *p* = 0.06, *η_p_* = 0.054. Non-significant interactions were reported for time by condition, GG_ms_ = 2.99, *F*_(1.89,113.55)_ = 0.144, *p* > 0.05, *η_p_* = 0.032, time by fitness, GG_ms_ = 1.89, *F*_(1.89,113.55)_ = 0.947, *p* > 0.05, *η_p_* = 0.016, and time by condition by fitness, GG_ms_ = 0.367, *F*_(1.89,113.55)_ = 0.244, *p* > 0.05, *η_p_* = 0.004 (see Figure 2).

### Heart Rate

**Figure 3:**
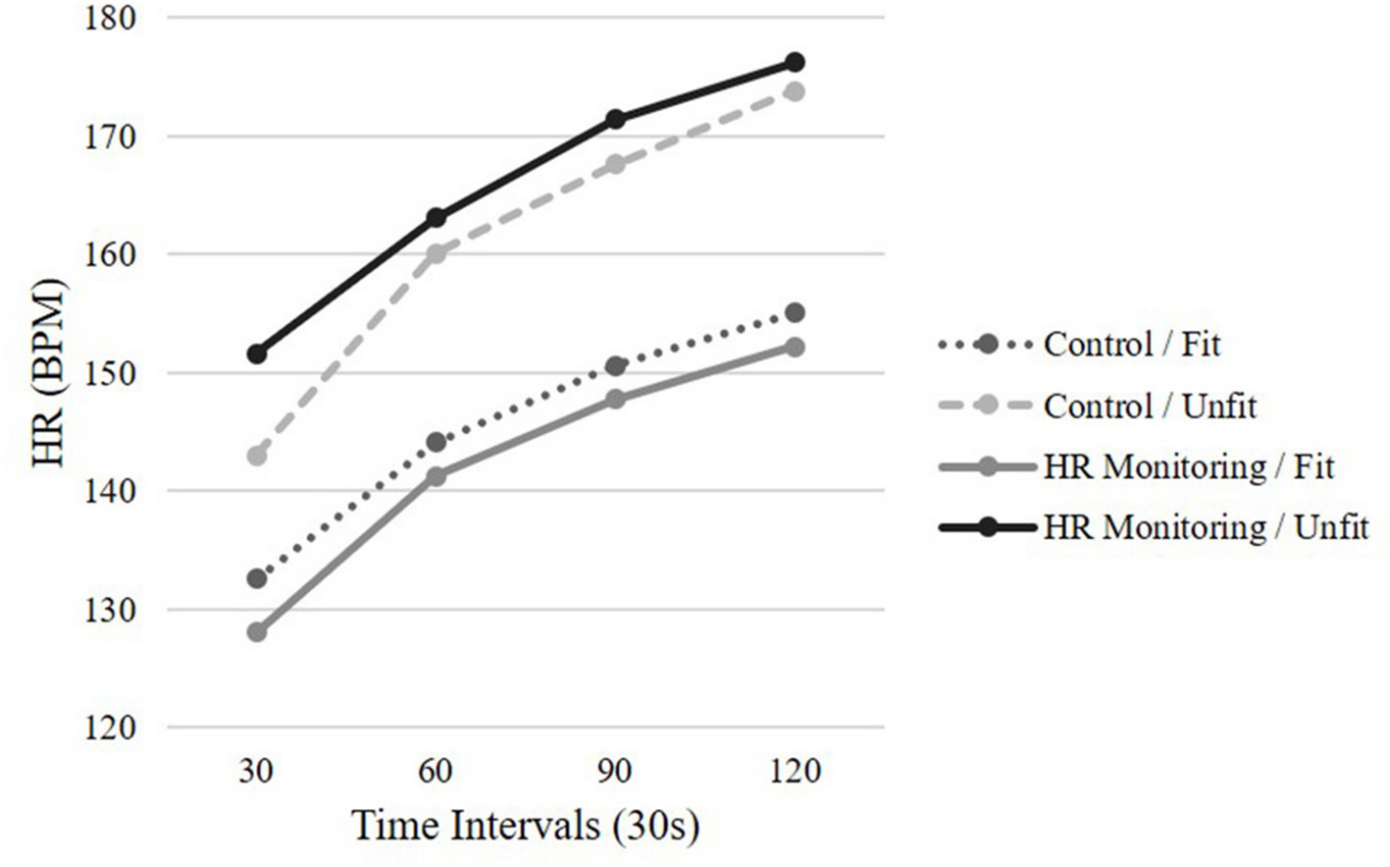
Mean HR across time by condition and fitness levels.

The analysis conducted on HR was similar to the one conducted on RPE and attention. A significant main effect of time was revealed, GG_ms_ = 14524.61, *F*_(1.51,90.64)_ = 372.55, *p* < 0.001, *η_p_* = 0.861. This suggests that HR increased with time across the conditions and fitness levels. The main effect for fitness was significant, *F*_(1,60)_ = 40.73, *p* < 0.001, *η_p_* = 0.40, indicating that unfit participants had higher HR compared to fit participants. There was no significance in time by condition, GG_ms_ = 29.195, *F*_(1.511,90.64)_ = 0.749, *p* > 0.05, *η_p_* = 0.441, time by fitness, GG_ms_ = 104.316, *F*_(1.511,90.64)_ = 0.2.676, *p* > 0.05, *η_p_* = 0.043, or time by condition by fitness, GG_ms_ = 100.395, *F*_(1.511,90.64)_ = 2.575, *p* > 0.05, *η_p_* = 0.041 (see Figure 3).

## Discussion

With the rapid increase in WFD, which includes HR monitors, more research is needed to examine the effects of HR monitoring on the perception of exertion and attention allocation during an exertive stepping task with individuals of varying fitness levels. Contrary to expectations, RPE was not higher for unfit individuals nor the condition with HR monitoring. This aligns with previous research reporting that RPE is not influenced by fitness levels (Parfitt et al., 1996; Faulkner and Eston, 2007; Faulkner et al., 2007). However, other previous research has reported that fit individuals exhibit lower RPE compared to unfit individuals (Hassmen, 1990; Travlos and Marisi, 1996). In the current study, unfit individuals reported significantly lower RPE levels at the 30-s time point compared to fit individuals. This may have occurred because the unfit individuals have less experience with exercise and RPE which could result in inaccurate RPE levels. With abstract concepts, like exertion, adults rely on available contextual information from prior knowledge (Borghi et al., 2011). However, as time progressed RPE increased to similar levels of fit individuals at the 90 and 120-s time points. All participants' RPE ratings increased linearly with time and effort which is in line with previous research (Hutchinson and Tenenbaum, 2007; Basevitch et al., 2011; Ritchie et al., 2016). No significant difference was reported between the two conditions (e.g., HR monitoring vs. control). HR monitoring does not influence RPE regardless of fitness level. It was hypothesized that HR monitoring would shift attention toward the physiological cues thus increasing RPE levels, however, this was not supported. This demonstrates that all individuals regardless of fitness level can monitor their HR to guide physical activity training without influencing RPE. HR monitoring allows for intervention designs to track exercise intensity and increase PA (Dooley et al., 2017).

Having knowledge of HR during the task diverted the attention of the participants across both fitness levels, rejecting the hypothesis. These results align with the parallel processing model (Rejeski, 1985) which suggests a limited channel capacity through which information can pass from perceptual field to focal awareness. Therefore, only a limited number of sensory cues may be brought into consciousness at one time. Consequently, both internal and external cues could compete for attention (Pennebaker and Lightner, 1980; Stones, 1980). In the current study, the external stimuli of the HR monitor could have been cognitively appealing for dissociation to occur (Dyrlund and Wininger, 2008). Moreover, it is plausible that the HR monitor provided some distraction which could have allowed the participants to dissociate from the aversive stimuli posed by the stepping task. As the stepping task became more difficult, the participants' attention become more associative and they may be focusing on the exertive sensations (Hutchinson and Tenenbaum, 2007; Basevitch et al., 2011; Ritchie et al., 2016).

There are several limitations to this study. First, there were unequal numbers of fit and unfit individuals as well as the majority of participants being female. Also, the mean age of participants was ~22 years old. Therefore, future research should have equal number of participants in each fitness level and gender as well as examine younger and older populations. Furthermore, future research should consider more categories for fitness levels. Another limitation is the unequal recovery time between the two tasks. The unfit individuals took longer to return to resting HR levels which could have influenced fatigue during the experimental task. Future research should utilize a standard recover time period. Additionally, only HR was monitored. Other research should include the different aspects of WFD such as accelerometry, steps, and calories burned as well as other WFD devices that are reliable and valid. Results can only be applied to an exertive stepping task in a laboratory setting. Other exercise modalities should be examined to determine if the results are similar in a free-living environment.

The current findings have relevance for applied practitioners implementing PA interventions with individuals who monitor their HR. With the increasing rates of physical inactivity and obesity, it is important to help promote active and healthy lifestyles. WFD are helpful for practitioners and individuals to monitor their PA levels. Self-monitoring is an effective behavioral intervention in that observing one's own behavior may produce change in the desired direction (Cooper et al., 2007). Monitoring HR has shown to increase overall PA and moderate-to-vigorous PA (McManus et al., 2008). Practitioners can use these WFD for interventions to increase PA and PA intensity. Having an individual monitor their HR at 30-s intervals while performing an exertive task may help dissociate attention while not influencing their exertion levels.

In conclusion, monitoring one's HR while performing an exertive stepping task diverts attention externally regardless of fitness level. However, no RPE differences were reported between the two conditions. Despite having higher HRs, unfit individuals reported significantly lower levels of RPE during the first time point compared to fit individuals. The results of this study demonstrate that self-monitoring HR can be effective in PA interventions from a psychophysiological perspective.

## Data Availability Statement

The raw data supporting the conclusions of this article will be made available by the authors, without undue reservation.

## Ethics Statement

The studies involving human participants were reviewed and approved by Texas Christian University IRB. The participants provided their written informed consent to participate in this study.

## Author Contributions

RB-T, AW, and KH: conceptualization. RB-T, AW, KH, EW, and JR: methodology, writing—review, and editing. RB-T: formal analysis, writing—original draft preparation, and supervision. All authors have agreed to the published version of the manuscript.

## Funding

This work was supported in part by grants from the Texas Christian University Junior Faculty Summer Research Program and open access library funds.

## Conflict of Interest

The authors declare that the research was conducted in the absence of any commercial or financial relationships that could be construed as a potential conflict of interest.

## Publisher's Note

All claims expressed in this article are solely those of the authors and do not necessarily represent those of their affiliated organizations, or those of the publisher, the editors and the reviewers. Any product that may be evaluated in this article, or claim that may be made by its manufacturer, is not guaranteed or endorsed by the publisher.
